# Molecular biomarkers for Alzheimer’s disease and related dementias – improved methods to facilitate global studies

**DOI:** 10.1002/alz.087345

**Published:** 2025-01-09

**Authors:** Henrik Zetterberg

**Affiliations:** ^1^ Institute of Neuroscience and Physiology, The Sahlgrenska Academy at University of Gothenburg, Mölndal, Sweden; UCL Institute of Neurology, Queen Square, London United Kingdom

## Abstract

**Background:**

Recent advancements in sample collection and storage methods, as well as biomarker measurement technologies, have made it possible to determine highly accurate biomarkers for Alzheimer’s disease (AD) in regular plasma samples collected by venipuncture or as dried plasma spots.

**Methods:**

A novel ultrasensitive biomarker measurement method called NUcleic acid Linked Immuno‐Sandwich Assay (NULISA), which improves the sensitivity of traditional proximity ligation assays to attomolar level, by suppressing assay background via a dual capture and release mechanism, was evaluated against Single molecule array (Simoa) technology for AD biomarker measurement. Dried plasma spots were evaluated in relation to regularly collected EDTA plasma samples.

**Results:**

Proteins from dried plasma spots, stored at room temperature over weeks, can be eluted and AD‐related biomarkers, including P‐tau217, neurofilament light (NfL) and glial fibrillary acidic protein (GFAP), can be accurately quantified with r values above 0.80 (p<0.00001) compared with validated Simoa‐based measurements. Absolute concentrations of biomarkers in dried plasma spots are lower than in regularly collected venous samples. NULISA was at least 10‐fold as sensitive to measure AD‐related biomarkers compared with Simoa; a multiplexed NULISA method allows for the measurement of P‐tau217, NfL and GFAP concentrations in a single blood drop. Data from under‐represented populations will be presented.

**Conclusion:**

The measurement of biomarkers in dried plasma spots using novel ultrasensitive measurement technologies is feasible for biomarker assessment in remote populations with limited access to healthcare resources. Dried plasma spot‐specific reference limits are needed. Combining this with digital assessment tools for cognitive function should facilitate studies of the global epidemiology of dementia.

